# Human Exposure Assessment of Indoor Dust: Webster and Stapleton Respond

**DOI:** 10.1289/ehp.1206470R

**Published:** 2013-04-01

**Authors:** Thomas F. Webster, Heather M. Stapleton

**Affiliations:** 1 Department of Environmental Health, Boston University School of Public Health, Boston, Massachusetts; 2 Nicholas School of the Environment, Duke University, Durham, North Carolina, E-mail: heather.stapleton@duke.edu

We agree with Cao et al. that methods for sampling dust are insufficiently uniform between research groups and can be improved (Allen 2008a; Harrad et al. 2010). By using refined dust sampling methods we should be able to reduce exposure measurement error, likely random, leading to increased associations with exposure biomarkers. We have conducted several studies on polybrominated diphenyl ethers (PBDEs) investigating methods of dust sampling, the relationship between dust concentrations and potential sources of PBDEs, dust concentrations and biomarkers of exposure, and the use of handwipes as an intermediary step (Allen et al. 2008a, 2008b; Stapleton et al. 2008; Watkins et al. 2011, 2012; Wu et al. 2007). It is worth noting that dust sampling for environmental chemicals can have several purposes, including exposure assessment and characterization of sources. Dust sampling is also subject to a number of practical constraints such as sampling logistics and the requirement for sufficient mass of dust for adequate quantification of target compounds. We believe hand-wipes represent a more biologically relevant measure of indoor exposure for PBDEs than dust sampled from the floor of a room. Handwipes may also represent exposure experienced by direct contact with PBDE-treated sources. In addition, handwipes may integrate exposure across multiple microenvironments (Watkins et al. 2011, 2012). We agree that the dust particle size is likely to play a role in exposure to PBDEs, and this factor has received relatively little attention in the past. Recent work by Weschler and Nazaroff (2010) suggests that, on average, semi-volatile organic compounds (including relatively more volatile pentaBDE congeners) are distributed in a room between air, dust, and surface films roughly as expected by equilibrium partitioning. The levels of pentaBDEs in all of these sampling media are therefore likely to show associations with body burden, although refinement of sampling methods may improve associations. The situation may be different for BDE-209, the main constituent of decaBDE that is essentially non-volatile at room temperature. It may escape from products via friability rather than volatilization (Webster et al. 2009). The particle size distribution of BDE-209 in dust may be different than that of pentaBDEs. Finally, researchers and risk assessors estimate exposure to chemicals in dust by multiplying dust concentrations by highly uncertain exposure factors for dust ingestion (U.S. Environmental Protection Agency 2011). Additional research on dust ingestion factors is needed.

## References

[r1] Allen JG, McClean MD, Stapleton HM, Webster TF (2008a). Critical factors in assessing exposure to PBDEs via house dust.. Environ Int.

[r2] Allen JG, McClean MD, Stapleton HM, Webster TF (2008b). Linking PBDEs in house dust to consumer products using X-ray fluorescence (XRF).. Environ Sci Technol.

[r3] Harrad S, Abdallah M, de Wit C, Östman C, Bergh C, Covaci A (2010). Indoor contamination with hexachloro-cyclo-dodecanes, polybrominated diphenyl ethers, and perfluoro-alkyl compounds: an important exposure pathway?. Environ Sci Technol.

[r4] Stapleton HM, Kelly SM, Allen JG, McClean MD, Webster TF (2008). Measurement of polybrominated diphenyl ethers on hand wipes: estimating exposure from hand to mouth contact.. Environ Sci Technol.

[r5] U.S. Environmental Protection Agency (2011). Exposure Factors Handbook: 2011 Edition. EPA/600/R-09/052F. Washington, DC:U.S. Environmental Protection Agency.. http://www.epa.gov/ncea/efh/pdfs/efh-complete.pdf.

[r6] Watkins DJ, McClean MD, Fraser AJ, Weinberg J, Stapleton HM, Sjödin A (2011). Exposure to PBDEs in the office environment: evaluating the relationships between dust, handwipes, and serum.. Environ Health Perspect.

[r7] Watkins D, McClean M, Fraser A, Weinberg J, Stapleton HM, Sjödin A (2012). Impact of dust from multiple microenvironments and diet on pentaBDE body burden.. Environ Sci Technol.

[r8] Webster TF, Harrad S, Millette JR, Holbrook RD, Davis JM, Stapleton HM (2009). Identifying transfer mechanisms and sources of decabromodiphenyl ether (BDE 209) in indoor environments using environmental forensic microscopy.. Environ Sci Technol.

[r9] Weschler CJ, Nazaroff WW (2010). SVOC partitioning between the gas phase and settled dust indoors.. Atmos Environ.

[r10] Wu N, Herrmann T, Paepke O, Tickner J, Hale R, Harvey E (2007). Human exposure to PBDEs: associations of PBDE body burdens with food consumption and house dust concentrations.. Environ Sci Technol.

